# Electrical stimulation‐based nerve location prediction for cranial nerve VII localization in acoustic neuroma surgery

**DOI:** 10.1002/brb3.981

**Published:** 2018-05-04

**Authors:** Dilok Puanhvuan, Sorayouth Chumnanvej, Yodchanan Wongsawat

**Affiliations:** ^1^ Department of Biomedical Engineering Faculty of Engineering Mahidol University Nakhon Pathom Thailand; ^2^ Surgery Department Faculty of Medicine Ramathibodi Hospital Mahidol University Bangkok Thailand

**Keywords:** acoustic neuroma, CMAP, CN VII localization, electrical stimulation

## Abstract

**Introduction:**

Cranial nerve (CN) VII localization is a critical step during acoustic neuroma surgery because the nerve is generally hidden due to the tumor mass. The patient can suffer from Bell's palsy if the nerve is accidentally damaged during tumor removal. Surgeons localize CN VII by exploring the target area with a stimulus probe. Compound muscle action potentials (CMAPs) are elicited when the probe locates the nerve. However, false positives and false negatives are possible due to unpredictable tissue impedance in the operative area. Moreover, a single CMAP amplitude is not correlated with probe‐to‐nerve distance.

**Objectives:**

This paper presents a new modality for nerve localization. The probe‐to‐nerve distance is predicted by the proposed nerve location prediction model.

**Methods:**

Input features are extracted from CMAP responses, tissue impedance, and stimulus current. The tissue impedance is calculated from the estimated resistance and capacitance of the tissue equivalent circuit. In this study, experiments were conducted in animals. A frog's sciatic nerve and gastrocnemius were used to represent CN VII and facial muscle in humans, respectively. Gelatin (2.8%) was used as a mock material to mimic an acoustic neuroma. The %NaCl applied to the mock material was used to emulate uncontrollable impedance of tissue in the operative area.

**Results:**

The 10‐fold cross‐validation results revealed an average prediction accuracy of 86.71% and an average predicted error of 0.76 mm compared with the measurement data.

**Conclusion:**

The proposed nerve location prediction model could predict the probe‐to‐nerve distance across various impedances of the mock material.

## 1 INTRODUCTION

Vestibular schwannoma, also known as acoustic neuroma, is a benign tumor initiating from the vestibular nerve the so‐called cranial nerve (CN) VIII. The tumor grows in the cerebellopontine angle area, causing stretching and suppression of the surrounding cranial nerves (trigeminal, cochlear, and facial nerves) and the brainstem. Postoperative facial nerve palsy can possibly occur if the facial nerve or CN VII is accidentally damaged.

Preserving CN VII during acoustic neuroma surgery is a critical concern (George, 2001; Kartush, Graham, Bouchard, & Audet, 1991; Møller, 2011; O'Brien, 2008). Reliable nerve localization techniques can aid in preventing permanent nerve damage. Intraoperative monitoring systems (IOMs) have become the standard for intracranial surgeries and are used to monitor biological signals and to apply external stimulation. Furthermore, continuous free‐running electromyography (EMG) of the facial muscles can be monitored. Mechanical irritation including touching, pulling, stretching, and compressing the CN VII causes bursts and spikes in EMG activity. The surgeon is alerted of the CN VII location by audible spikes and bursts (Johann, Christian, & Rudolf, 2000; Kombos et al., 2000; Prass & Lüders, 1986). Nevertheless, mechanical‐based nerve stimulation can damage the nerve. Preoperative nerve localization can be performed using magnetic resonance imaging (MRI). Diffusion tensor tracking (DTT) or high‐density diffusion tensor imaging (HD‐DT) can be applied to MRI to localize the target nerve (Roundy, Delashaw, & Cetas, 2012; Zhang et al., 2013), and thus, the surgeon can prelocalize the CN VII location prior to surgery. However, this technique is not real‐time because the nerve becomes dislocated due to anatomical changes during acoustic neuroma surgery. Nerve localization can be performed by applying an energy, that is, magnetic, acoustic, or electric power to the nerve. Compound muscle action potentials (CMAPs) will be elicited at some latency poststimulation. Given the high rate of change in magnetic fields, eddy currents can be induced in the target tissue (Barker, Jalinous, & Freeston, 1985). This technique is known as transcranial magnetic stimulation (TMS), which is noninvasive nerve stimulation. Target tissue at a focal point approximately 2–3 mm beneath the stimulus coil is remotely stimulated (Ueno, Tashiro, & Harada, 1988), and TMS can be used to localize the motor cortex. However, TMS has less localization resolution due to the large focal area. Miniature TMS is feasible, but focal depth and the induced eddy current are relatively small (Bonmassar et al., 2012). Large magnetic fields can interfere with other instruments in the operating room. A recent study demonstrated that applying high‐intensity focused ultrasound (HIFU) below the ablation level could modulate the motor cortex (Kim, Chiu, Lee, Fischer, & Yoo, 2014; Yoo et al., 2011). Acoustic waves noninvasively travel through nontarget tissue and formulate high acoustic pressure at the focal point. Tail and leg movements are elicited when the focal point is located in the motor areas of rats (Kim et al., 2014) and rabbits (Yoo et al., 2011). HIFU stimulation has high nerve localization resolution due to its small focal point. However, fewer studies focusing on neuro modulation have been reported, and no study has succeeded in activating CMAPs by stimulation of the sciatic nerve or other nerve routes (Bystritsky et al., 2011; Colucci, Strichartz, Jolesz, Vykhodtseva, & Hynynen, 2009). Laser stimulation is also feasible for nerve localization (Teudt, Nevel, Izzo, Walsh, & Richter, 2007; Wells et al., 2005). A high nerve localization resolution can be achieved using a fine laser beams, although the nerve must be exposed to the laser light. Electrical stimulation is used in conventional CN VII localization in acoustic neuroma surgery. IOM is currently acknowledged as the standard for nerve preservation during an operation (George, 2001; Møller, 2011; O'Brien, 2008). IOM supports electrical stimulation and biological signal monitoring. Nerve can be localized by applying current pulses to the target area. A CMAP response will be elicited if the nerve receives a sufficiently stimulus current. This technique is characterized by a high localization resolution. The surgeon can explore the nerve location by positioning the tip of the probe at the suspected area. CMAP amplitude can be used for CN VII preservation: a 50% reduction in CMAP amplitude compared with baseline is considered an unfavorable criterion for surgical intervention to prevent poor postoperative outcome (Amano et al., 2011). Constant current stimulation is generally used rather than constant voltage control in nerve localization applications because voltage is driven to overcome tissue impedance. Mono polar probe configuration exhibit shows superior results to bipolar probe configurations, as the current can penetrate deeper into the target area (Kartush et al., 1991). However, false positives and false negatives can possibly occur due to uncontrollable impedance in the operative area. In practice, the stimulus current is fixed at some intensity (0–2 mA). Current may be lost to low impedance tissue, such as cerebrospinal fluid (CSF) or normal saline (Møller, 2011), which is known as “current shunting current.” In addition, current can jump from high impedance tissue such as tumors to nearby nerve tissue, which is termed “current jump.” CMAP responses indicate only the nerve activation process. A single CMAP amplitude is not correlated with the distance between the probe and the nerve, and the exact distance from the probe's tip to the nerve remains unknown.

This paper aimed to develop a nerve location prediction system for IOM during acoustic neuroma surgery. A new modality of CN VII localization is proposed herein. The probe‐to‐nerve distance is estimated by the proposed nerve location prediction model. This model is formulated based on various assumptions. Input features are extracted from CMAP responses, the impedance of the target area, and the stimulus current. Rather than considering a signal CMAP, multi‐CMAP responses are analyzed to represent linear model. Thus, new method of impedance measurement is proposed, allowing, impedance variations of tissues in the operative area to be observed.

## 2 MATERIALS AND METHODS

**Figure 1:**
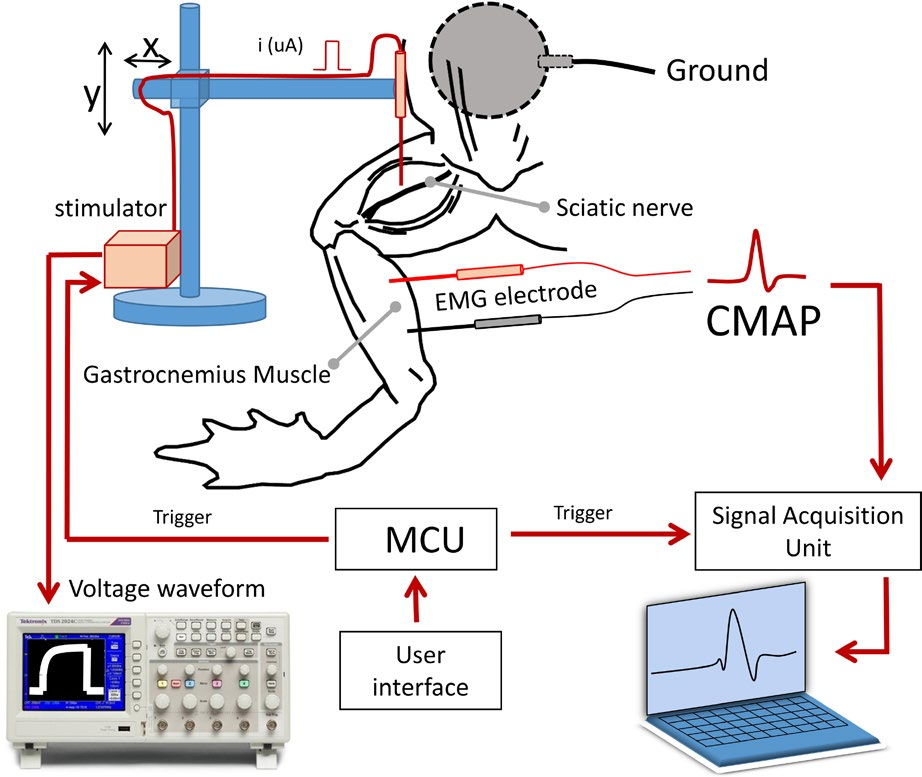
Experimental setup

A frog's sciatic nerve and gastrocnemius muscle were used to represent CN VII and facial muscle in humans. Figure 1 shows the experiment setup. The sciatic nerve is exposed to the stimulus probe mounted on an adjustable 2 degree‐of‐freedom (x‐ and y‐axis) holder. The probe position can be adjusted with a resolution of 100 us. Electrical stimulation is initiated by user command. When the microcontroller unit (MCU) receives a stimulation command from the user, it sends a trigger pulse to the stimulator to generate a current pulse through the probe. The trigger signal is sent to the signal acquisition unit as a marker for CMAP segmentation in the further analysis. The voltage waveform of the current pulse is previewed on an oscilloscope. The CMAP response is captured by a pair of EMG needle electrodes inserted in the gastrocnemius muscle. All signals, including the voltage waveform, the trigger, and the EMG signal containing the CMAP responses, are monitored and recorded by a computer.

### 2.1 Animal preparation

All applicable international, national, and institutional guidelines for the care and use of animals were followed. All procedures performed in studies involving animals were in accordance with the ethical standards of the Mahidol University Animal Care and Use Committee (COA No. MU‐ACUC 2014/001). Frogs are generally used in neural experiments because they have long sciatic nerves and large gastrocnemius muscles that can generate high amplitude signals by a brief electrical pulses. Both frog's sciatic nerve and human's CN VII are myelinated nerves and have synapses. Furthermore, the size of frog's sciatic nerve is comparable to human's CN VII. Human's CN VII has 1.18 ± 0.31 mm diameter (Xu et al., 2009) where frog's sciatic nerve diameter is approximately 1 mm (Peled, Cory, Raymond, Kirschner, & Jolesz, 1999). Hence, in this study, the CN VII and facial muscle in humans were represented by a frog sciatic nerve and gastrocnemius muscle, respectively. The frogs were prepared in the following steps (Elaine & Marieb, 2010; Pal & Pal, 2005); (1) The frog was stunned to induce unconsciousness by striking below the head. (2) The unconscious frog was gently held to locate the depression point at the connection of the skull and the vertebral column. (3) A needle was inserted to penetrate the skin, muscle, and bone to the spinal cord. (4) The frog underwent a pithing process during which the frog's brain and cord were damaged by manipulating the needle anterior into the skull and posterior into the spinal cord. Using this procedure, the frog remains alive but losses its voluntary movement and other reflexes. (5) The skin was cut and removed from the middle trunk of the frog. (6) The muscle was separated to expose the sciatic nerve at the thigh. (7) The nerve and muscle was moistening with Ringer's solution (0.6% NaCl, 0.014% KCl, 0.012% CaCl2, 0.02% NaHCO3, and 0.1% dextrose).

### 2.2 Electrical nerve stimulation and signal acquisition

Figure 1 shows the experiment setup of this study. A Digitimer DS3 (Digitimer Ltd, Hertfordshire, UK) current‐controlled (CC) stimulator was used to apply a monophasic electrical current pulse to the sciatic nerve. This CC stimulator has four current ranges allowing precise control of output current between 2 μA and 32 mA with adjustable pulse widths in the range of 10 us–1000 ms. Current pulses of the desired intensity and pulse width were delivered using a Standard Prass Flush‐Tip Monopolar Stimulator Probe (Medtronic, MN, USA). A 1.20‐inch surface diameter ground electrode (MFI Medical Equipment Inc., CA, USA) was placed on the frog's abdominal wall. Electric pulses were initiated by a trigger signal from the microcontroller in the trigger unit. The driven voltage waveform of the current pulse was captured using a Tektronix TDS2004B digital oscilloscope (Tektronix Inc., OR). This waveform was acquired by OpenChoice Desktop software (Tektronix Inc). The electric pulses initiate action potentials (APs) in the sciatic nerve, which induce muscle twitching. Muscle contractions cause the generation of a CMAP. The CMAP can be captured by a twisted pair of subdermal needle electrodes (SGM Medical, Split, Croatia). A single subdermal needle electrode (SGM Medical, Split, Croatia) was used as a ground electrode and inserted in the gastrocnemius tendon. EMG signals were amplified using a BIOPAC MP100 amplifier (BIOPAC systems Inc, USA). The gain and sampling frequency were 1000 and 2 kHz, respectively. The trigger signal generated by the microcontroller was also acquired through a digital input pin of the BIOPAC MP100. During the experiment, EMG signals containing CMAP responses and trigger pulses were continuously monitored and recorded by AcqKnowledge 3.9.1 software (BIOPAC systems Inc). The CMAP responses occur in the EMG signal at some latency after the trigger pulse.

### 2.3 Impedance measurement

Impedance is an essential predictor of nerve localization. Low impedance leads to shunting current, causing false‐negative nerve localization. High impedance leads to current jump from high to low impedance, causing false‐positive nerve location prediction. From the literature, several bio‐impedance measurement techniques have been proposed to solve this issue. By containing the target tissue within a cylindrical tube of known size and applying a certain voltage, the impedance can be calculated from the current and the known cross‐sectional area (*A*) and length (*l*) (Kyle et al., 2014) of the cylinder. However, this technique may not be suitable for acoustic neuroma surgery because the target area cannot be formulated into a specific cylindrical shape. Impedance of human tissue can be measured in terms of dielectric properties (Marsland & Evans, 1987) by delivering high‐frequency sweep signal (GHz) to the target tissue through a coaxial probe. This technique requires network analyzer to determine the conductivity and permeability of the tissue. Bioelectrical impedance analyzer was previously developed as an integrated circuit (IC) AD5933 (Analog Device Corp., MA, USA) (Ferreira, Seoane, Ansede, & Bragos, 2010; Ferreira, Seoane, & Lindecrantz, 2011) that can be interfaced with a microcontroller. It acquires a midfrequency range (kHz) of a certain voltage to the target tissue and converts the frequency response to impedance by discrete time Fourier transform. A three‐electrode configuration is applicable for tissue impedance determination (Morimoto et al., 1993; Organ, Tasker, & Moody, 1968). The RC components in the tissue equivalent circuit can be estimated from the pulse responses captured by the inner electrode in the three‐electrode setup.

**Figure 2:**
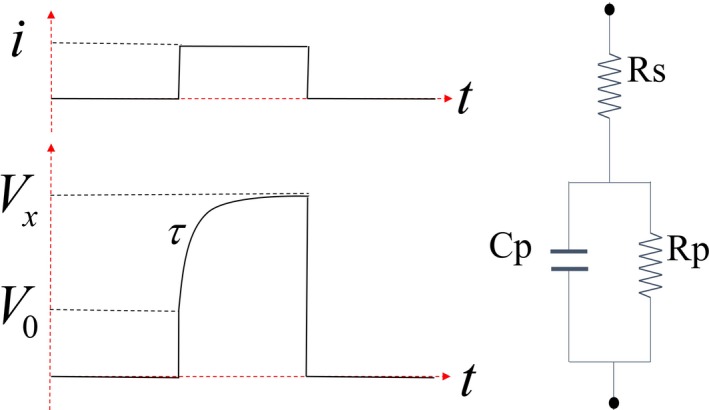
Typical current and voltage waveforms (left) applied to the tissue equivalent circuit (right)

**Figure 3:**
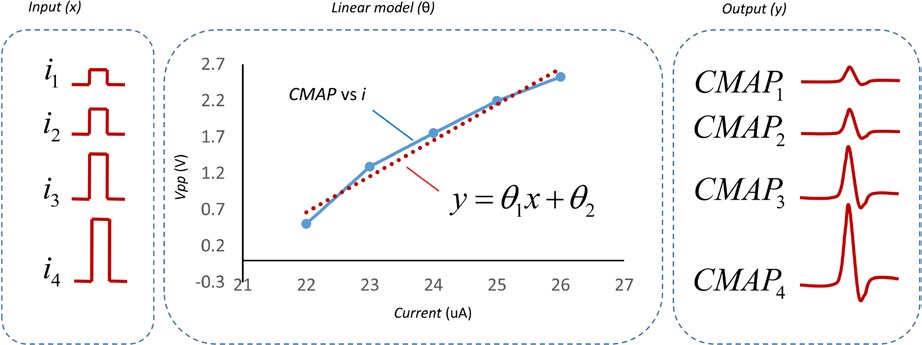
Linear model of multi‐CMAP responses for multiple stimulus inputs in one trial

In this paper, tissue impedance is calculated from the value of the RC component in the tissue equivalent circuit. Figure 2 (right) shows the equivalent circuit of normal tissue, which includes *R*_s_, *C*_p_ and *R*_p_. (Aroom et al., 2009; Webster, 2009). Figure 3 (left) shows typical current (*i*) and voltage waveforms (*V*) applied to the equivalent circuit. Because the stimulator generates a constant current, current *i* is constant during stimulation. The pulse response represented as a voltage waveform (*V*) is varied depending on the *R*_s_, *C*_p_ and *R*_p_ values. Therefore, these RC components can be calculated from prior knowledge of *i* and *V*. Because *C*_p_ is a short circuit at *t*_*0*_, *R*_s_ can be calculated from equation (1). *R*_p_ can be determined when *C*_p_ is fully charged at *t*_x_ by equation (2).

(1) $$R_{s}=\frac{V_{0}}{i}$$

(2) $$R_{p}=\frac{V_{x}}{i}-R_{s}$$

where *V*_*0*_ is the voltage at the rising edge at *t*_*0*_ and *V*_*x*_ is the saturated voltage at *t*_*x*_.

*C*_*p*_ is calculated from the time constant (τ) by equation (5). τ was estimated by nonlinear least‐square curve fitting as shown in equation (3) (Dennis, 1977). The voltage curve in the period of *t*_*0*_ to *t*_*x*_ is fit by equation (4). The function *F*(τ*,t*) was modeled based on the assumption of the voltage waveform (*V(t)*) across the RC component of the tissue equivalent circuit.

(3) $$\tau =\min_{\tau }\sum_{i=t_{0}}^{t_{x}}{\left(F\left(\tau ,t_{i}\right)-V_{i}\right)}^{2},$$

(4) $$F\left(\tau ,t\right)=V_{0}+\left[\left(V_{x}+V_{0}\right)\times \left(1-e^{-\frac{t}{\tau }}\right)\right],$$

(5) $$C_{p}=\frac{\tau }{R_{p}}.$$

### 2.4 Preparation of the mock material

In this study, we used 1.4% gelatin (weight/volume) (Gelita, Germany) as mock material to mimic the mechanical and electrical properties of brain tissue (Mobashsher & Abbosh, 2014). Nevertheless, different tissues and liquids that have different electrical properties may be present in the operative area. To imitate impedance variation in operative area, thus, the impedance of the mock material should be varied by adding NaCl (Kandadai, Raymond, & Shaw, 2012). In this experiment, we used 2.8% gelatin to mimic brain tumor as it has a higher density than normal brain tissue.

Four different mock materials were formulated in this experiment. Impedance was varied from high to low by varying the % NaCl. 2.8% gelatin with 0% NaCl was labelled Mat_1_ as the highest impedance material. The impedances were lower for Mat_2_, Mat_3_ and Mat_4_, corresponding to 0.25%, 0.5% and 0.75% NaCl, respectively.

### 2.5 Linear nerve‐muscle modeling

A CMAP response is elicited when current is injected into the nerve. Hence, probe position according to CMAP output is denoted as the CN VII location. However, the CMAP amplitude is not correlated with the distance between the probe and the nerve. Electric current may be lost or resisted by low or high impedances of the mediated material, such as tumor or tissue, CSF and normal saline. Moreover, CMAP amplitude is subject dependent due to different muscle volumes. Hence, analysis of single CMAP is not reliable for predicting nerve location.

From explicit nerve‐muscles stimulation, motor output represented by CMAP amplitude increases in accordance with stimulus intensity (Colletti, Fiorino, Policante, & Bruni, 1997; McComas, Fawcett, Campbell, & Sica, 1971; Tokimura et al., 2014). Greater stimulus intensities allow more electrical current to penetrate through the axon in the nerve trunk, causing motor recruitment, as shown in Figure 3. This paper represents the nerve‐muscle response as a simple linear model denoted by the linear equation, *y* = θ_1_ *x* + θ_2_, where *x* is the varied stimulus intensity (*i*_1−4_), *y* is the peak‐to‐peak voltage (*V*_pp._) of CMAP responses (CMAP_1−4_), and θ is a parameter of the linear model. To eliminate subject‐dependent issues, CMAP responses were normalized (NCMAP) in particular trails by equation (6).

(6) $${\text{NCMAP}}_{i}=\frac{{\text{CMAP}}_{i}-{\text{CMAP}}_{1}}{{\text{CMAP}}_{4}-{\text{CMAP}}_{1}}\times 100,$$

where *i* is varied according to the stimulus current level from 1 to 4.

The linear equation parameters (θ_1_, θ_2_) in each stimulus trial can be calculated by the linear least‐square solution (Douglas, Montgomery, & Geoffrey, 2012) as shown in equation (7).

(7) $$\theta ={\left(X^{T}X\right)}^{-1}X^{T}y,$$

where $\theta =\left[\begin{array}{c} \theta_{1}\\ \theta_{2} \end{array}\right]$, $X=\left[\begin{array}{cc} 1 & i_{1}\\ \vdots & \vdots \\ 1 & i_{4} \end{array}\right]$ , $y=\left[\begin{array}{c} {\text{NCMAP}}_{1}\\ \vdots \\ {\text{NCMAP}}_{4} \end{array}\right]$.

The θ_1_ and θ_2_, slope and offset of linear model parameters, were used as a representative nerve‐muscle response.

### 2.6 Experimental procedure

Twelve sciatic nerves were included in this experiment. Each sciatic nerve underwent with following procedures;

1. The tip of the stimulus probe is positioned on the sciatic nerve by adjusting the adjustable platform.
2. This probe location is defined as zero distance (*d *=* *0).
3. The tip of the probe is advanced upward approximately 30 mm
4. The mock material Mat_1_ is placed over the sciatic nerve
5. The tip of the probe is adjusted to the zero‐distance position.
6. A small current pulse is applied, and the stimulus intensity is gradually increased until CMAPs appear in the EMG signal.
7. This stimulus intensity is recorded as the motor threshold (MT) or $i_{1}^{0}$ at *d *=* *0.
8. The driven voltage waveform of $i_{1}^{0}$ is recorded and labelled *V* ^*0*^
9. Four pulses of $i_{1}^{0}$ with interstimulus interval (ISI) were approximately 500 ms are applied to obtain a grand averaged ${\text{CAMP}}_{1}^{0}$
10. Four pulses of 110%MT are applied and labelled $i_{2}^{0}$ to obtain the grand averaged ${\text{CAMP}}_{2}^{0}$
11. Four pulses of 120%MT are applied and labelled $i_{3}^{0}$ to obtain the grand averaged ${\text{CAMP}}_{3}^{0}$
12. Four pulses of 130%MT are applied and labelled $i_{4}^{0}$ to obtain the grand averaged ${\text{CAMP}}_{4}^{0}$
13. The probe tip is moved 1 mm upward, denoted *d *=* *1.
14. Steps 6–12 are repeated to obtain $i_{1-4}^{1}$, ${\text{CAMP}}_{1-1}^{1}$and *V* ^*1*^.
15. The probe tip is moved 1 mm upward, denoted *d *=* *2.
16. Steps 6–12 are repeated to obtain $i_{1-4}^{2}$, ${\text{CAMP}}_{1-4}^{2}$and *V* ^*2*^.
17. The probe tip is moved 1 mm upward, denoted *d *=* *3.
18. Steps 6–12 are repeated to obtain $i_{1-4}^{3}$, ${\text{CAMP}}_{1-4}^{3}$and *V* ^*3*^.
19. The probe tip is moved 1 mm upward, denoted *d *=* *4.
20. Steps 6–12 are repeated to obtain $i_{1-4}^{4}$, ${\text{CAMP}}_{1-4}^{4}$and *V* ^*4*^.
21. All steps 3–20 are repeated for each mock material, Mat_2,_ Mat_3_ and Mat_4_

### 2.7 Nerve location prediction model

**Figure 4:**
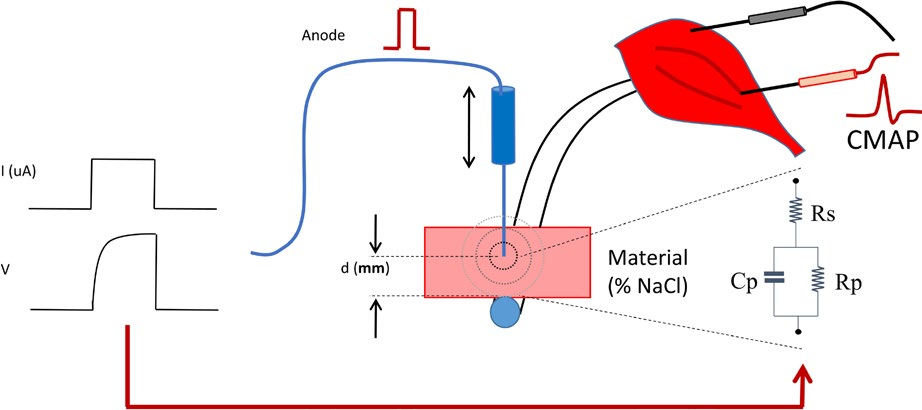
Diagram of the probe‐to‐nerve distance prediction

This experiment was performed to imitate a real acoustic neuroma surgical environment. Figure 4 shows a diagram of the probe‐to‐nerve distance prediction experiment. The frog's sciatic nerve was hidden by the mock tissue material. The impedance of the mock material was varied by %NaCl. The distance (*d*) between the probe's tip and the nerve trunk was adjusted and labelled as an output feature. Input features were extracted from the stimulus current, the impedance, and CMAP responses. To find the distance of the nerve beneath the probe, two assumptions were formulated.

In the first assumption, when the probe contacts to the nerve, the CMAP amplitude is elicited after the stimulus onset. The CMAP amplitude is increased proportionally with the stimulus intensity. Therefore, the CMAP amplitude (*CMAP*) depends directly on the stimulus current (*i*_MT_) and the proportional gain (λ_1_). According to the experiment setup shown in Figure 4, when the probe is located at some distance from the nerve, the CMAP amplitude would also be decreased due to leakage of the stimulus current and the resistance according to the probe‐to‐nerve distance (*d*) and the impedance of mediated tissue (*Z*). Hence, CMAP amplitude (CMAP) could be modelled calculated by equation (8),

(8) $$\text{CMAP}=\lambda_{1}\frac{i_{\mathrm{MT}}}{d\times Z}+\eta_{1},$$

where *i*_MT_ is the MT, or the minimum stimulus current that can activate CMAP, *d* is the probe‐to‐nerve distance, *Z* is impedance of the material between the probe and the nerve, λ_*1*_ is a multiplier, and η_1_ is the error.

In the second assumption, the CMAP latency, (the delay of CMAP peak after the stimulus pulse) depends on the length of the nerve root. When the probe is contacted to the nerve, given a stimulus pulse, the action potential takes some certain time propagating along the nerve to the motor. In this study, the delay would be increased when the probe is located at some distance (*d*) from the nerve trunk. Moreover, different materials between the probe and the nerve which have different electrical permeability and conductivity would influence the pulse delay. Hence, the CMAP latency (*t*_*L*_) depends directly on the distance (*d*) and the impedance (*Z*) of material between the probe and the nerve. CMAP latency can be modelled by equation (9),

(9) $$t_{L}=\lambda_{2}\left(Z\times d\right)+\eta_{2},$$

where λ_2_ is a multiplier and η_2_ is the error.

To estimate the probe‐to‐nerve distance, equations (8) and (9) are combined, as given by equation (10).

(10) $$d=\lambda_{1}\frac{i_{\mathrm{MT}}}{\text{CMAP}\times Z}+\lambda_{2}\frac{t_{L}}{Z}+\eta ,$$

where η is the total error η_1_+ η_2_

Let λ be an array of multiregression model parameters, λ = [λ_1_, λ_2_, η]. *X* is a matrix input data set, where X = $\left[\frac{i_{\mathrm{MT}}}{\text{CMAP}\times Z},\frac{t_{L}}{Z},1\right]$. *d* is the output data set calculated by a function *F*(λ*, X)*. The multiregression model parameters (λ) can be estimated by nonlinear least‐squares fitting, as shown in equation (11) [Dennis, 1977].

(11) $$\lambda =\min_{\lambda }\sum_{i}{\left(F\left(\lambda ,X_{i}\right)-d_{i}\right)}^{2}.$$

The proposed nerve location prediction model would be validated by 10‐fold cross‐validation.

## 3 RESULTS

**Figure 5:**
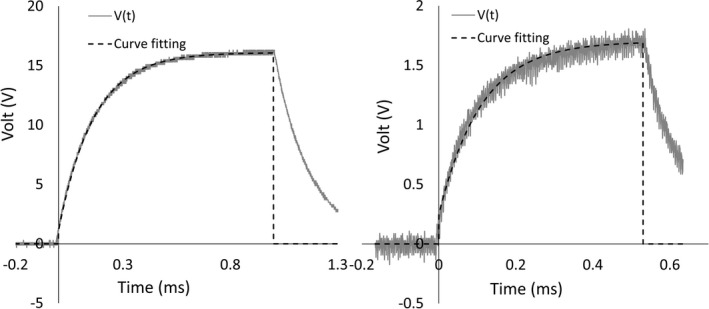
The captured voltage waveforms (V(t)) that were applied to real resistors and capacitors of the tissue equivalent circuit (left graph, gray line) and the frog's sciatic nerve (right graph, gray line). Dashed lines show curve fitting with V(t)

*R*_s_, *R*_p_, and *C*_p_ in the tissue equivalent circuit were calculated from the driven voltage waveform (*V(t)*) of current pulse. First, the proposed impedance measurement was tested with well‐known resistors and capacitors. Figure 5. (left) shows *V(t)* across the equivalent circuit with *R*_s_ = 1 kΩ, *R*_p_ = 15 kΩ, and *C*_p_ = 10 nF. The current intensity was 1 mA and, *R*_s_, *R*_p_, were calculated from equations (1) and (2), respectively, and *C*_p_ was determined by the time constant (τ) according to equation (5). τ was estimated from curve fitting based on equations (3) and (4) in Figure 5, and the dashed line of the estimated τ was correlated with *V(t)* with *R*‐squared = 0.99. The results show that *R*_*s*_, *R*_p_, and *C*_p_ were 1.09 kΩ, 15.00 kΩ, and 10.82 nF, respectively. The proposed impedance measurement method accurately estimates resistors and capacitor connected as the tissue equivalent circuit (Figure 2 (right)).

Figure 5 (right) shows *V(t)* captured during stimulation of the frog's sciatic nerve. Curve fitting (dashed line) was correlated with *V(t)* with *R*‐squared = 0.97. The current intensity was 30 uA. The results show that *R*_s_, *R*_p_, and *C*_p_ of the sciatic nerve were 8.21 kΩ, 48.48 kΩ, and 2.27 nF, respectively.

The impedances of the mock materials (Mat_1−4_) were measured. The electrical intensity was adjusted to 250 uA. Table 1 shows the *R*_*s*_, *R*_*p*_, and *C*_*p*_ that were calculated from the proposed method. Impedance *Z* was calculated from the RC equivalent circuit at frequency = 500 kHz. The result shows that the resistances (*R*_s_, *R*_p_) and impedance (*Z*) decreased when %NaCl was increased, whereas, capacitance (*C*_p_) increased.

**Table 1:** Example of impedance measurements of 2.8% gelatin with varied %NaCl

Name	% NaCl	*R‐*square	*R*_s_ (Ω)	*R*_p_ (Ω)	*C*_p_ (nF)	*Z* at 500 kHz
Mat_1_	0%	0.89	1087.36	19520.00	0.82	1161.44
Mat_2_	0.25%	0.96	858.11	7360.00	5.67	860.37
Mat_3_	0.50%	0.97	577.51	6720.00	7.83	579.19
Mat_4_	0.75%	0.98	330.75	6480.00	10.39	332.31

**Figure 6:**
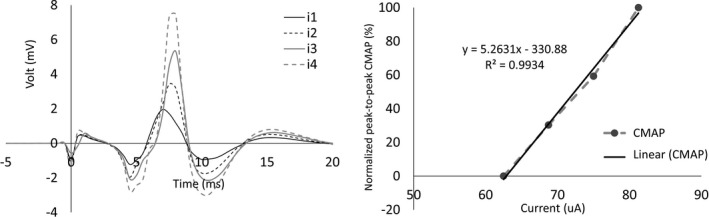
(Left) example of CMAP responses (CMAP1‐4) from the varied stimulus intensities (i1‐4), (Right) linear model that is used to represent CMAP output

CMAP amplitude increased when the stimulus intensity was increased. Figure 6 (left) shows an example of CMAP responses the highest stimulus current (*i*_*4*_) generated the largest CMAP amplitude (gray dashed line). Figure 6 (right) shows the linear relationship between stimulus intensities and the normalized peak‐to‐peak voltage of CMAP responses (*NCMAP*_*i*_). Linear equation parameters (θ_1_, θ_2_) were determined by equation (7). The results show that the linear model parameters θ_1_ and θ_2_ of the example in Figure 6 were 5.26 and −330.88, respectively. The average CMAP latency was 7.25 ms. This latency changed with distance and mock material used.

**Figure 7:**
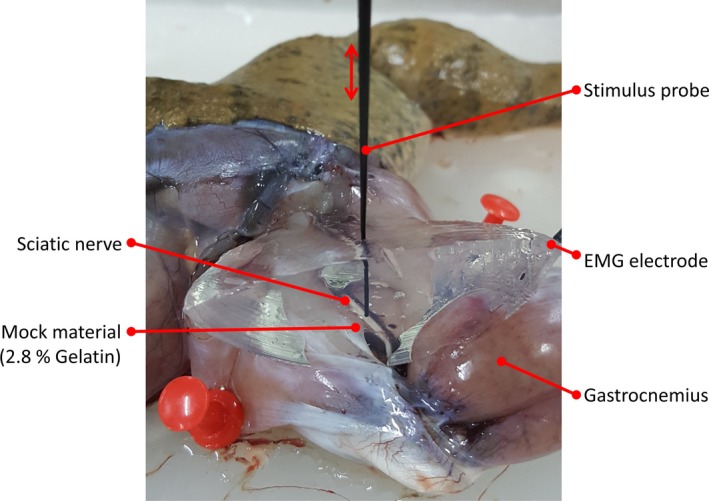
Nerve localization experiment. This photograph was captured when the stimulus probe was adjusted to zero distance from the frog's sciatic nerve. The mock material was placed above the sciatic nerve

Figure 7 shows an example of a nerve localization experiment. These data were prepared after completing experiments for twelve frog sciatic nerves. The voltage waveforms according to stimulus intensities of each trial were analyzed to estimate the impedance (*Z*) and RC values (*R*_*s*_, *R*_*p*_, and *C*_*p*_) in the equivalent circuit. CMAP responses elicited in EMG signals were segmented and labelled according to trigger pulses. Grand averaged CMAP responses (CMAP_1−4_) were calculated according to each stimulus pulse (*i*_1−4_). The peak‐to‐peak voltage of the grand averaged *CMAP*_1−4_ was normalized and used as input features for linear nerve model estimation. The linear model parameters θ_*1*_ and θ_*2*_ are used to represent the CMAP responses. CMAP latency (*t*_*L*_) was obtained by measuring the delay of the CMAP peak after the trigger. The average CMAP latency of each trial was calculated over four grand averaged CMAP responses (CMAP_1−4_).

**Figure 8:**
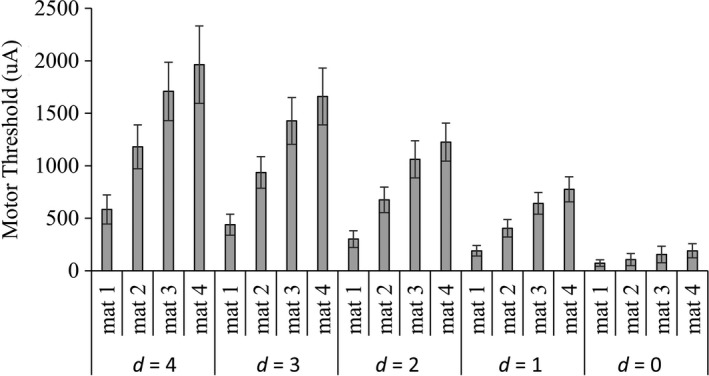
Motor threshold (*i*_MT_) of all sciatic nerve (*n *= 12) in different probe‐to‐nerve distance (*d *=* *0–4 mm) and mock material (mat1‐mat4)

**Figure 9:**
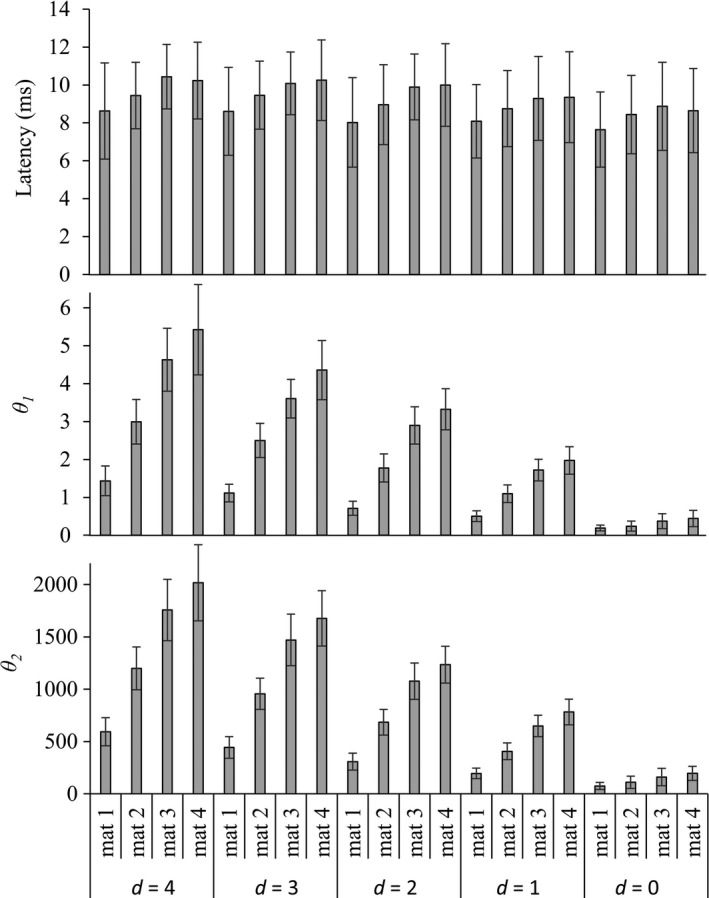
CMAP response, latency (*t*_*L*_), and linear equation parameter (θ_*1*_ and θ_*2*_) of all sciatic nerve (*n *= 12) in different probe‐to‐nerve distance (*d *=* *0–4 mm) and mock material (mat1‐mat4)

**Figure 10:**
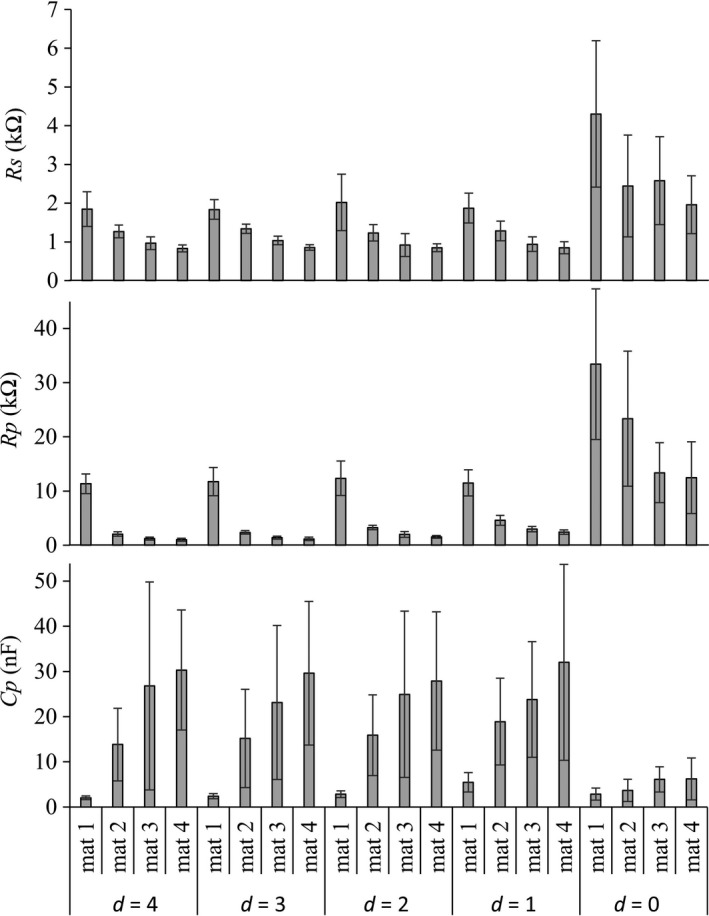
RC equivalent circuit parameters (*R*_s_ *, R*_p_, and *C*_p_) of all sciatic nerve (*n *= 12) in different probe‐to‐nerve distance (*d *=* *0–4 mm) and mock material (mat1‐mat4)

Figures 8, 9, and 10 show the experimental data of the motor threshold (*i*_*MT*_), CMAP response (*t*_*L,*_ θ_1_ and θ_2_) and impedances of RC network parameters (*R*_s_ *, R*_p_, and *C*_p_), respectively, of all 12 subjects in different probe‐to‐nerve distance (*d *=* *0–4 mm) and mock material (*mat1‐mat4*). According to Figure 8, motor threshold was increased when the probe‐to‐nerve distance was increased. With the same probe‐to‐nerve distance, mock material with high %Nacl required higher current to induce motor response.

According to Figure 9, similar pattern to motor threshold data could be observed in the linear equation parameters data (θ_1_ and θ_2_). CMAP latency tended to increase with the increasing probe‐to‐nerve distance and %NaCl. According to Figure 10, *R*_s_ and *R*_p_ were decreased, whereas *Cp* was increased when %NaCl is increased. When the stimulus probe contact with the nerve trunk, *R*_s_ and *R*_p_ were higher where *C*_p_ was lower than the probe‐to‐nerve distance greater than zero.

In total, 240 data sets were obtained from 12 frog sciatic nerves using four mock materials (*mat1‐mat4*) and five distances (*d *=* *0−4 mm). Input features including impedance parameters (*Z*,* R*_s_, *R*_p_ and *C*_p_,), motor threshold (*i*_MT_), and CMAP response parameters (*t*_*L*_, θ_1_ and θ_2_) were extracted and used to calculate the multiregression parameters (λ) by equation (11). The proposed nerve location prediction model shown in equation (10) was validated by 10‐fold cross‐validation. The average error was 1.6 mm (*SD *= 0.74 mm, *n *=* *10) compared with the measured data (*SD* represents standard deviation). The average prediction accuracy was 37.14% (*SD* = 0.18 %, *n *=* *10), and prediction accuracy was calculated from the correlation coefficient of the prediction results and the test set.

The prediction results were enhanced by modification of error (η_1_, η_2_) in equation (8) and (9). Equation (12) shows the modified error η_1_. Equation (8) is based on the relationship of CMAP amplitude with the stimulus current, distance, and impedance. Hence, the η_1_ error function is composed of the associated parameters shown in *X*_1_.

(12) $$\eta_{1}=\left[\sum_{j=0}^{4}b_{1}\left(j\right)X_{1}\left(j\right)\right],$$

where, *X*_1_(0) = 1, *X*_1_(1) = θ_1_, *X*_1_(2) = θ_2_, X_1_(3) = R‐squared of curve fitting in the linear nerve model, *X*_*1*_(*4*)* *= $\frac{i_{\mathrm{MT}}}{R_{p}}$, and *b*_*1*_ is the multi‐regression model parameter.

Equation (13) shows the modified error η_2_. Equation (9) is based on the relationship of CMAP latency with distance and impedance. Hence, the η_2_ error function is composed of the associated parameters shown in *X*_2_ and *R*.

(13) $$\eta_{2}=\left[\sum_{j=0}^{3}b_{2}\left(j\right)X_{2}\left(j\right)+\sum_{k=0}^{1}\alpha \left(k\right)e^{\beta (k)R(k)}\right],$$

where *R*(0)  = *R*_s_, *R*(1)  = *R*_p_, *X*_2_(0)  = *C*_p_, *X*2(1)  = R‐squared error of τestimation, *X*_2_(2)  = $\frac{R_{s}}{C_{p}}$, *X*_2_(3)  = *t*_L_, and parameters b2, α and β are multi‐regression model parameters.

**Figure 11:**
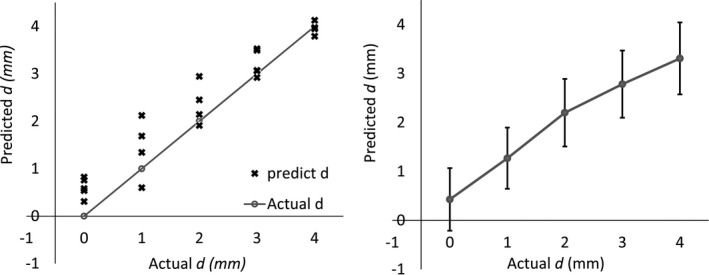
Example of onefold in 10‐fold cross‐variation or probe‐to‐nerve distance prediction output (left) and average (*n *=* *48) probe‐to‐nerve distance (*d *=* *0–4) prediction of all 10‐fold cross‐validation results (right)

The nerve location prediction model as show in equation (10) was modified with the new error function (equation (12), (13)). The multiregression model parameters were recalculated and validated with 10‐fold cross‐validation. The results revealed that the average error was enhanced from 1.6 mm (*SD* = 0.74 mm, *n *=* *10) to 0.76 mm (*SD* = 0.15 mm, *n *=* *10). The average prediction accuracy was increased from 37.14% (*SD* = 0.18 %, *n *=* *10) to 86.71% (*SD* = 0.07 %, *n *=* *10). Figure 11 (left) shows an example of the probe‐to‐nerve distance prediction output of onefold in 10‐fold cross‐validation. The predicted probe‐nerve distance (**×** symbol) was linearly correlated with the actual distance (gray line). Figure 11 (right) shows the average (*n *=* *48, 12 sciatic nerve with four mock materials) probe‐to‐nerve distance (*d *=* *0–4) prediction of all 10‐fold cross‐validation results (right). The predicted probe‐to‐nerve distance results illustrate linear relationship with actual data.

## 4 DISCUSSIONS

The proposed impedance measurement method was performed based on an estimation of the RC components from the driven voltage waveform. The RC values in the equivalent circuit were accurately predicted in a real resistor and capacitor network. The driven voltage waveform of a constant current pulse use to stimulate a frog's sciatic nerve is similar to a real RC circuit. The RC components in the equivalent circuit of the frog's sciatic nerve and the mock material could be estimated. The impedance (*Z*) of some specific frequency (*Hz*) could be calculated based on the RC values in the tissue equivalent circuit. The estimated impedance of the frog sciatic nerve is comparable to that reported in previous research (Organ et al., 1968; Morimoto et al., 1993). Varying %NaCl in the mock material effects the estimated impedance value (Kandadai et al., 2012). Therefore, a wide range of impedances of the mock material (*Mat*_1−4_) can be used to represent uncontrollable tissue impedances in the operative area.

This impedance measurement method is applicable to actual acoustic neuroma surgeries. Voltage waveforms commonly occur during constant current pulse stimulation. The tissue impedance was immediately estimated after applying a current pulse. From the literature, an additional frequency sweep (Aroom et al., 2009; Ferreira et al., 2010; Marsland & Evans, 1987) three‐electrode configuration (Organ et al., 1968; Morimoto et al., 1993) is required for bio‐impedance analysis. Hence, existing methods may not be suitable for clinical use in real acoustic neuroma surgeries, as they require additional stimulation and specific probe configuration.

Upon delivering a certain stimulus intensity to a target area, a CMAP response is elicited if the nerve is located in the suspected area. In practice, a fixed high stimulus current of 0–2 mA is used for CN VII exploration (George, 2001; Møller, 2011; O'Brien, 2008). However, CMAP responses may disappear if the stimulus current is lost or resisted by surrounding tissue, known as “current shunting,” or “current jump” (Møller, 2011). Hence, monitoring a single CMAP response is not reliable. Moreover, CMAP amplitude is subject dependent and is not correlated with the distance between the probe tip and nerve trunk.

This study presents the nerve‐muscle output via a linear model. Multi‐CMAP responses were obtained for varied stimulus intensities. The first stimulus intensity (*i*_1_) was the lowest current that could elicit a CMAP signal. This stimulus intensity is termed motor threshold (MT). The MT was then increased in increment of 10% corresponding to *i*_2−4_. CMAP amplitude was increased according to stimulus intensity. CMAP amplitude is related to motor unit recruitment, which is dependent on the number of axons activated. Increasing the stimulus intensity caused more current to penetrate the nerve bundle. The peak‐to‐peak voltage of CMAP responses had a linear relationship with the stimulus intensity. Hence, a linear equation is feasible to represent the nerve‐muscle output. Moreover, a linear model was formulated from the normalized CMAP responses and stimulus intensities of each trial. Therefore, the problem of subject dependence could be neglected. The estimated linear model parameters (θ_1_, θ_2_) were used as representative nerve‐muscle output. Shifting the linear model parameters is feasible for predicting nerve function (Puanhvuan, Chumnanvej, & Wongsawat, 2013). A search algorithm for *i*_*1*_ can be used to eliminate the lost or resisted currents flowing into the nerve. However, maximum stimulus current must not exceed safety criteria. The linear model parameters (θ_1_, θ_2_) would be related to the nerve location.

Probe‐to‐nerve distance was estimated by the proposed multiregression model. Regression parameters were calculated from the input data set including CMAP response (θ_1_, θ_2_, *t*_L_), impedance (Z) and stimulus current (*i*_MT_) parameters. Ten‐fold cross‐validation results showed high average error. The error function (η_1_, η_2_) could be modified in the proposed model to enhance the prediction results. An average prediction accuracy of 86.71% was achieved with a low average error of 0.76 mm. The estimated RC values in the tissue equivalent circuit are essential features, and the linear model parameters (θ_1_, θ_2_) were related to the probe‐nerve distance. The prediction error is acceptable because the predicted distance is linearly correlated with the measurement data. The nerve can be stimulated via the MT search algorithm. Finally, using this model, false positives and false negatives from current shunting and current jump would be decreased.

## 5 CONCLUSIONS

The experiment was conducted in an animal model. The sciatic nerve and gastrocnemius muscle of frogs were used to represent CN VII and facial muscle in humans, respectively. A mock material (2.8% gelatin) was used to mimic an acoustic neuroma. The %NaCl of the mock material was varied to model uncontrollable impedances of tissue in real operations. In practice, nerve localization of the CN VII is performed by the surgeon using an electrical probe to explore the target area. However, monitoring a single CMAP response elicited by a stimulus intensity is unreliable. A single CMAP amplitude is not correlated with probe‐to‐nerve distance.

This paper employed multi‐CMAP responses elicited by varying stimulus intensities for nerve‐muscle output modeling. The CMAP output was represented by linear model parameters (θ_1_, θ_2_). Moreover, other feasible predictors such as impedance and stimulus current were considered. Impedance at the stimulus area was determined from the estimated RC values of the tissue equivalent circuit from the driven voltage waveform analysis. Importantly, this impedance measurement technique does not require additional frequency sweeps or a specific electrode configuration.

In summary, the proposed multiregression model can be used for nerve location prediction to estimate the probe‐to‐nerve distance. All parameters, including motor response (θ_1_, θ_2_, *t*_L_), impedance (*Z*,* R*_s_, *R*_p_ and *C*_p_), and current pulse (*i*_MT_) parameters, were used as input features. The average prediction accuracy was 86.71%, and the average error was 0.76 mm. A prediction error of 0.76 mm is acceptable because the predicted distances were linearly correlated with the measured data. The nerve is highly likely to be stimulated via the MT search procedure. False positives and false negatives from current shunting and current jump are reduced using this method. The results obtained from the animal study demonstrate the feasibility of applying this technique to humans. Furthermore, the proposed nerve location prediction method does not require additional stimulation or other modules. Therefore, the proposed method could be incorporate into available IOM systems.
